# Making sense of response: How policies affect climate vulnerability

**DOI:** 10.1007/s13280-025-02140-w

**Published:** 2025-02-03

**Authors:** Alexandra Malmström, Janina Käyhkö, Aleksi Räsänen, Julia Tuomimaa, Sirkku Juhola

**Affiliations:** 1 Environment and Ecosystems Research Programme, University of Helsinki, Viikinkaari 1, Biocentre 3, 4414, 00790 Helsinki, Finland; 2 Natural Resources Institute Finland, Oulu, Finland; 3 Geography Research Unit, University of Oulu, P.O.Box 8000, 90014 Oulu, Finland

**Keywords:** Adaptation, Climate justice, Climate risk, Response, Vulnerability

## Abstract

There is a gap in understanding how different policies affect climate vulnerability and risk development, yet increasingly response is added to the risk framework. We propose a conceptual framework that explains how response and other policies affect risk determinants and demonstrate the application of the framework using a synthesis of empirical literature on climate-related health risks and adaptation in cities. The analysis shows that most of the policies affecting vulnerability and exposure are outside climate interventions, i.e., current conceptualization of response. The inclusion of response and other policies in risk assessments has implications for adaptation research and practice.

**Supplementary Information:**

The online version contains supplementary material available at 10.1007/s13280-025-02140-w.

## Concerns arising

Climate risk is the potential of adverse consequences for people or assets to occur, and it arises from the dynamic interactions between biophysical (hazards) and socio-economic (vulnerability, exposure, and ‘response’) determinants. In the context of climate change, ‘hazards’ include extreme weather and climate events, including both rapid and slow-onset, such as sea level rise, storms, heat waves, floods, for example (Möller et al. 2022). The importance of non-biophysical determinants of climate risk has been long recognized in the scientific community, mainstreamed in the 4th, 5th and 6th Assessment Reports and in the Special Report on Climate Extremes of the Intergovernmental Panel on Climate Change (Lavell et al. 2012; Oppenheimer et al. 2014; Ara Begum et al. 2022). These determinants include ‘exposure’ defined as the presence of people and assets in the locations and settings where they may get adversely affected, and ‘vulnerability’, which is defined as the predisposition of being adversely affected. Vulnerability is determined by a variety of personal and socio-economic factors, such as age, income, level of education, health status, among other (Möller et al. 2022). While studies have pointed out that vulnerability emerges through various social processes (Ribot 2014; Ford et al. 2018), a gap persists in understanding how various policies drive vulnerability over time (Jurgilevich et al. 2023).

The latest risk framework introduced in the IPCC 6th Assessment Report adds ‘response’ as one of the four determinants of climate risk, in addition to exposure, vulnerability, and hazard (Ara Begum et al. 2022). So far, response has been defined in the literature as climate interventions such as mitigation and adaptation, and the rationale of adding it as one of the four risk determinants is to better understand and account for the outcomes of climate interventions on hazard, vulnerability, and exposure (Ara Begum et al. 2022; Simpson et al. 2023). These outcomes may include intended outcomes in terms of risk reduction as well as unintended, both positive and negative (for example, maladaptation). Following the introduction of response into the mainstream climate risk assessment research (Simpson et al. 2021; Ara Begum et al. 2022), we hypothesize that the current conceptualization of response limited to climate interventions may not be sufficient to capture the emergence of vulnerability or to address its root causes. Hence, we identify three concerns, which need to be addressed to advance and link the research on vulnerability, risk, and response.

First concern is related to the understanding of *how* risk and vulnerability emerge and its implications for adaptive action. It is evident that risks are complex, have different time horizons, manifest through different pathways and are influenced by a vast range of dynamic social processes and policies beyond climate interventions, i.e. responses (Morris et al. 2017; Jurgilevich et al. 2023). The complexity is a challenge for adaptation planning in general, and in particular, in avoiding maladaptation (Reckien et al. 2023) and disarray in policy agendas (Hedlund 2023).

Second concern is that limiting response to climate interventions risks excluding policies that moderate vulnerability (Jurgilevich et al. 2023) or are related to its root causes (Ribot 2014; Thomas et al. 2019), which has implications for justice. Vulnerability is largely shaped by a range of policies such as welfare and equity enhancing policies, social and health care (Thomas et al. 2019), as well as by the social and physical environment, which are in their turn, to a large degree a product of past policy decisions (Jurgilevich et al. 2023). The preference towards technological and infrastructural solutions will continue if adaptation is mainly focused on the reduction of hazard severity and exposure, while vulnerability reduction is mainly approached through adaptive capacity building measures (e.g. awareness campaigns) without addressing the root causes of vulnerability in the first place (Andrews et al. 2023). Such technological responses are at risk of shifting the focus away from critical and transformative approaches to vulnerability reduction (Amorim-Maia et al. 2022), and may exacerbate existing or create new injustices (Thomas et al. 2019), (see, for example, green gentrification (Anguelovski et al. 2018)). Equity and justice are not taken into account sufficiently in adaptation (Juhola et al. 2022) and the link between adaptation measures and vulnerability reduction is rather weak (Chu and Cannon 2021; Coggins et al. 2021).

Third concern relates to the divide between (1) normative and critical adaptation urging for addressing the root causes of vulnerability and taking systemic perspective and transformative approaches to adaptation (Amorim-Maia et al. 2022), on the one hand, and (2) solution-oriented research and practice of adaptation driven by the urgency of action and need for usable and useful science, on the other (Ara Begum et al. 2022). So far, vulnerability research has had little influence on adaptation decision-making (Ford et al. 2018), and separating response from other policies driving vulnerability emergence perpetuates this divide.

We suggest that there is a need to (1) examine more in-depth the concept of response and its capacity to explain risk emergence and development, and (2) explore how a more nuanced understanding of vulnerability and exposure emergence can be better incorporated into climate risk assessments and adaptive action. To do this, we propose a conceptual framework that includes other policies beyond climate interventions and explains their interaction with vulnerability, exposure, and hazard. We acknowledge the influence of response on all three risk determinants, as well as the multidirectional dynamics among all four determinants, but pay specific attention to policies affecting vulnerability and exposure due to the persisting gap and justice implications.

## Response and other policies in risk emergence

### Current response conceptualisation and operationalisation

Response was included into the latest IPCC AR6 risk framework as one of the four risk determinants and is defined as climate interventions such as mitigation and adaptation (Ara Begum et al. 2022). While response and intervention both refer to intentional action, it has not been clear thus far how they are defined in more specific, creating challenges for further operationalization in empirical work. We acknowledge that response as an intentional intervention includes both public policy responses and autonomous action by individuals, communities or businesses. Here, we specifically focus on public policies as we are interested in identifying and explaining more systemic societal impacts of policies in terms of creating or alleviating risks. We rely on the definition of public policy as actions that contain goals and means to achieve them, with the varying degree of goals’ identification, definition, articulation and justification, including decisions to act or not to act (Howlett and Cashore 2014).

While the rationale of response inclusion was to disentangle complex relationships between climate interventions and other risk determinants, the operationalization of response has so far focused on the outcomes for the overall risk reduction (Andrews et al. 2023). We argue, however, that we need a more nuanced exploration of risk emergence, as current response conceptualization may not be sufficient to capture how exposure and vulnerability develop in the first place, and what implications this has for adaptive action.

The inclusion of response into climate risk assessments is also significant considering the complex nature of risk. Risk complexity stems from the multiple time horizons, on the one hand, and from different risk types and their interactions with social-ecological systems, producing different outcomes, on the other (Ara Begum et al. 2022; Andrews et al. 2023). Climate change poses *direct* risks that are spatially and temporally proximal, as well as *deferred* risks that are postponed in time and space in relation to a climate event (Morris et al. 2017). Additionally, climate change can produce *indirect* risks that result from climate change-induced disturbances in ecological systems and transmitted to people through social-ecological interactions (Smith et al. 2014). All three types of risks are shaped by a multitude of different policies beyond climate interventions (Jurgilevich et al. 2023), having implications for risk assessment and for adaptive action.

### Identifying policies driving climate risk determinants

**Fig. 1:**
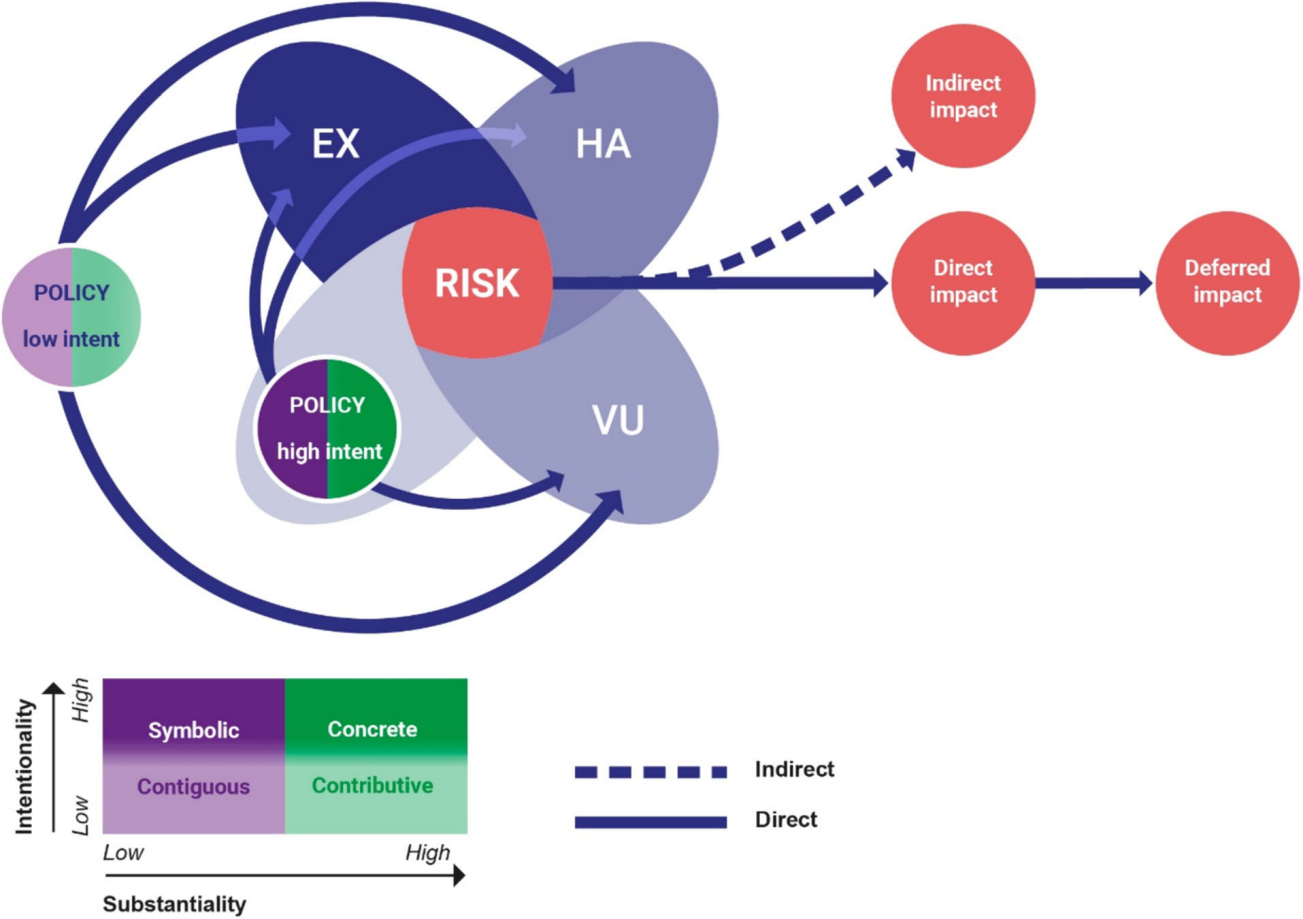
Conceptual framework of low and high intentionality policies (Dupuis and Biesbroek 2013) influencing risk determinants. High intentionality policies include climate interventions, so far conceptualized as response (Simpson et al. 2021). Risk further manifests in three types of impacts. These impacts include (1) direct—immediate and proximal, (2) indirect—following ecological disturbances and realized through the social-ecological interactions, and (3) deferred—postponed in time and/or space (based on Smith et al. 2014). *VU*  vulnerability, *EX * exposure, *HA * hazard

We propose a conceptual framework that expands the current understanding of response by considering also policies driving vulnerability and exposure in addition to the climate policy interventions (Fig. 1). To identify the relevant policies, we propose to use an analytical lens of intentionality and substantiality (Dupuis and Biesbroek 2013). Substantiality refers to the “contribution of a policy to reducing climate change vulnerability or benefit from climate change opportunities”, and intentionality to “the extent to which policies are purposefully designed or changed to manage the impacts of climate change, reduce vulnerability or enhance adaptive capacity” (Dupuis and Biesbroek 2013). These two dimensions split into ‘high’ and ‘low’ yield four policy categories: symbolic, concrete, contributive and contiguous policies (Table 1). We illustrate the categories with the help of policies related to the health risks of heat and flood as these are the most common risks in cities (Dodman et al. 2023).

**Table 1:** Four policy categories based on intentionality and substantiality (Dupuis and Biesbroek 2013), and guiding questions for the identification

Policy category	Intentionality	Substantiality	Guiding questions	Examples (for urban health risks)
Symbolic	High	Low	What are the first-order policies, i.e., responses (adaptation with high intentionality targeting vulnerability, exposure, or hazard)?	Adaptation strategies setting agenda and course of action (if no actions are defined)
Concrete	High	High		Policies with specific adaptation measures and actions: adaptation plans detailing actions, nature-based solutions, district cooling
Contributive	Low	High	What are the root-causes of vulnerability and exposure?	Urban planning policies directing urban form and pattern, disaster risk management
Contiguous	Low	Low	What are the moderating factors of vulnerability and exposure?	Air quality programs reducing UHI effect, public and occupational healthcare, social care policies
			What are the relevant policies related to root causes and moderating factors?	

S*ymbolic* policies (high intentionality and low substantiality) are typically those designed for adaptation but having no concrete effects on reducing vulnerability, exposure, or hazard. Depending on the content, symbolic policies can include, for example, adaptation strategies provided their focus is on setting the agenda, shaping the course of action and public discourse. It is important to note that often adaptation strategies and plans are used interchangeably to describe policies and programmes containing also specific measures, which are then better described as concrete. The agenda-setting policies are critical in creating space for *concrete* policies, which have high substantiality and intentionality, e.g. specific adaptation policies, action plans and measures, such as nature-based solutions or district cooling, for example (Table 1).

*Contributive* policies (low intentionality and high substantiality) are not designed for adaptation or risk reduction, but contribute to it substantially, for example, urban planning policies directing the density of built environment, urban form, and pattern (Salata et al. 2017; Arifwidodo and Chandrasiri 2020; Venter et al. 2020) (Table 1). Finally, c*ontiguous* policies characterized by low substantiality and low intentionality include those policies that are not intended to tackle climate change risks but have a minor impact on it. For example, for urban health risks of climate change, air quality policies can be considered contiguous, as they enable UHI reduction to improve air quality, and simultaneously contribute to the reduction of heat risk (Stone 2005; Kinney 2018) (Table 1).

To identify relevant policies, we propose to structure the assessment based on intentionality, first identifying high and then low intentionality policies. High intentionality policies include adaptation and are further referred to as responses (Table 1). To identify policies with low intentionality, one needs to understand what the determinants of vulnerability and exposure in question are, since these are object- and hazard-specific. To identify these policies, we propose the examination of (1) exposure and vulnerability root causes, and (2) factors moderating vulnerability and exposure. The root causes of vulnerability have been largely explored by political economy and ecology literature (see e.g. Ribot 2014), and refer to the underlying societal, economic, and political structures and policies that shape people’s sensitivity and adaptive capacity (Kelman 2014; Anguelovski et al. 2018). Moderating factors are related to the physical and social environment, and different policies that increase or decrease the severity of an impact through vulnerability and exposure (Jurgilevich et al. 2023).

The proposed conceptual framework integrates the latest risk conceptualization including response (Ara Begum et al. 2022) with the policy categorization based on intentionality and substantiality (Dupuis and Biesbroek 2013) and three impact categories that aid in exploring the complexity and time horizons of climate impacts (Smith et al. 2014) (Fig. 1).

## Policy intentionality and interaction with risk determinants

We examine the contribution of high and low intentionality policies to vulnerability, exposure, and hazard with the example of climate-related health risks in cities (see Supplementary materials for methods & materials). The focus on climate change impacts on urban residents’ health is largely dictated by the fact that cities harbour large concentrations of people and assets, and most common impacts of climate change on people are impacts on health. Furthermore, urban environment presents an interesting case to explore the framework application because climate-related health risks in cities emerge through all three pathways (direct, indirect and deferred), and because urban environment in itself exacerbates or alleviates them (Jurgilevich et al. 2023). We first contextualize the analysis by summarizing health impacts according to the three pathways of their emergence. We then place the policies in three risk pathways, categorize them according to their substantiality and intentionality, and explore the policies from the perspective of their influence on risk determinants.

### Policies influencing health risks related to ambient temperature extremes

**Fig. 2:**
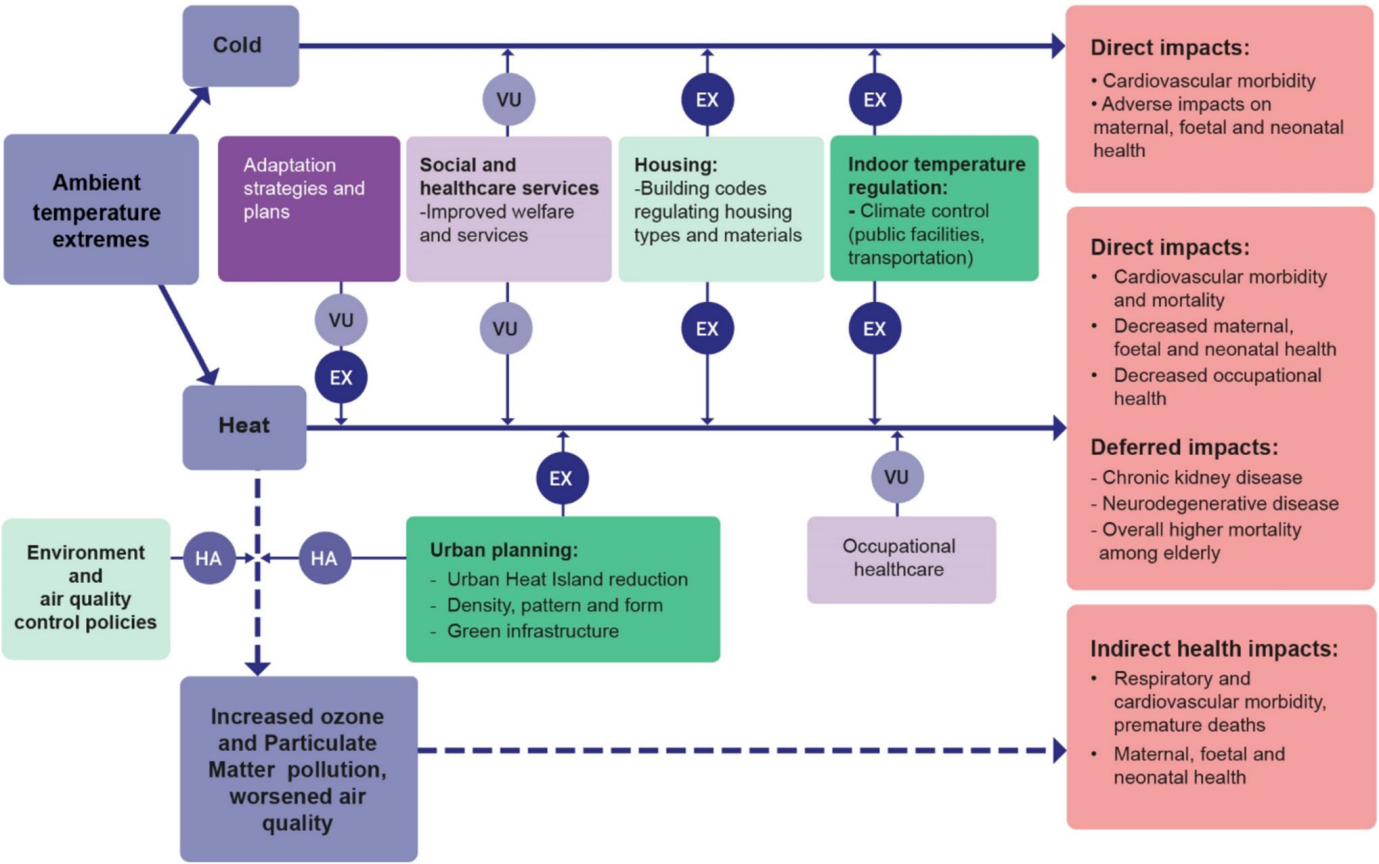
Ambient temperature extremes cause direct (mainly cardiovascular morbidity and mortality) and deferred (various chronic conditions) impacts on human health. Furthermore, heat and heatwaves exacerbate air quality in cities, causing indirect impacts on human health manifesting in, e.g. respiratory and cardiovascular morbidity, as well as in negative outcomes on maternal, phoetal and neonatal health. All three categories of impacts are influenced by policies with low and high intentionality. Dark purple denotes *symbolic* policies, dark green denotes *concrete* policies, fair purple denotes *contiguous* policies, fair green denotes *contributive* policies. *VU*  vulnerability, *EX * exposure, *HA* hazard. Dashed arrow denotes indirect impacts, solid arrow denotes direct and deferred impacts

Ambient temperature extremes impact human health in cities through three pathways (see for details Jurgilevich et al. 2023, and summary in Fig. 2). *Direct* impacts of extreme heat and heatwaves include negative outcomes for maternal, phoetal and neonatal health, occupational health (for outdoor workers), as well as overall increased preventable mortality and cardiovascular morbidity (see e.g. Kjellstrom 2016; Phung et al. 2016; Kuehn and Mccormick 2017). *Deferred* impacts, those that are postponed in time and/or space, are mainly related to prolonged heat exposure and chronic heat stress, and include various chronic health conditions, such as neurodegenerative diseases (Bongioanni et al. 2021) or chronic kidney diseases among outdoor workers (Tawatsupa et al. 2012). *Indirect* impacts of heat include those stemming from worsened outdoor and indoor air quality, as it is known that climate change and rising temperatures in cities affect ozone formation, spatial and temporal distribution of Particulate Matter (PM) and of airborne allergens (Kinney 2018). These ecological disturbances manifest in their turn in cardiovascular and respiratory morbidity, lung cancer, premature deaths and have negative effects on maternal, phoetal and neonatal health (Bernard et al. 2001; Lacasaña et al. 2005; Hiatt and Beyeler 2020).

#### High intentionality policies (responses)

*Symbolic* policies with high intentionality to reduce the impacts of ambient temperature extremes include adaptation strategies that set frameworks for concrete policies (Smith et al. 2014; Wong et al. 2021; Martín and Paneque 2022). Among the *concret*e policies, indoor temperature regulation in public places reduces *exposure* to both heat and cold risks (Carson et al. 2006). Additionally, urban planning can specifically aim at heat risk reduction (Arifwidodo and Chandrasiri 2020; Ellena et al. 2020). These policies intended to reduce citizens’ *exposure* to extreme heat have positive impacts on all heat-related health risks, including direct (CVD morbidity and mortality) (Sera et al. 2019; Ellena et al. 2020), deferred (Zanobetti et al. 2012; Shi et al. 2016), and indirect risks associated with worsened-air quality (Stone 2005) (Fig. 2).

#### Low intentionality policies

Policies with low intentionality tend to be focused on heat/cold *vulnerability* reduction, both through addressing the root causes of vulnerability, as well as moderating vulnerability (Fig. 2). More specifically, policies that influence citizens’ vulnerability are often *contiguous*, i.e., not necessarily labelled as adaptation, but contributing to reducing people’s *sensitivity* and increasing *adaptive capacity*. These include welfare, social and health care policies (Leichenko and Silva 2014; Rahman 2014). For example, occupational healthcare policies are *contiguous* in reducing *vulnerability* to the direct and deferred risks of heat (Tawatsupa et al. 2012; Kjellstrom 2016). In terms of indirect risks stemming from poor air quality, environment and air quality control policies have a positive impact on both air quality (*hazard*) directly, as well as on reducing the heat burden (Stone 2005; Kinney 2008). These policies are *contributive* to adaptation, since while they are not intended to enhance adaptation, they have a substantial contribution to reducing the severity of a cascading risk/hazard.

### Policies influencing health risks of floods, storms, and extreme precipitation

**Fig. 3:**
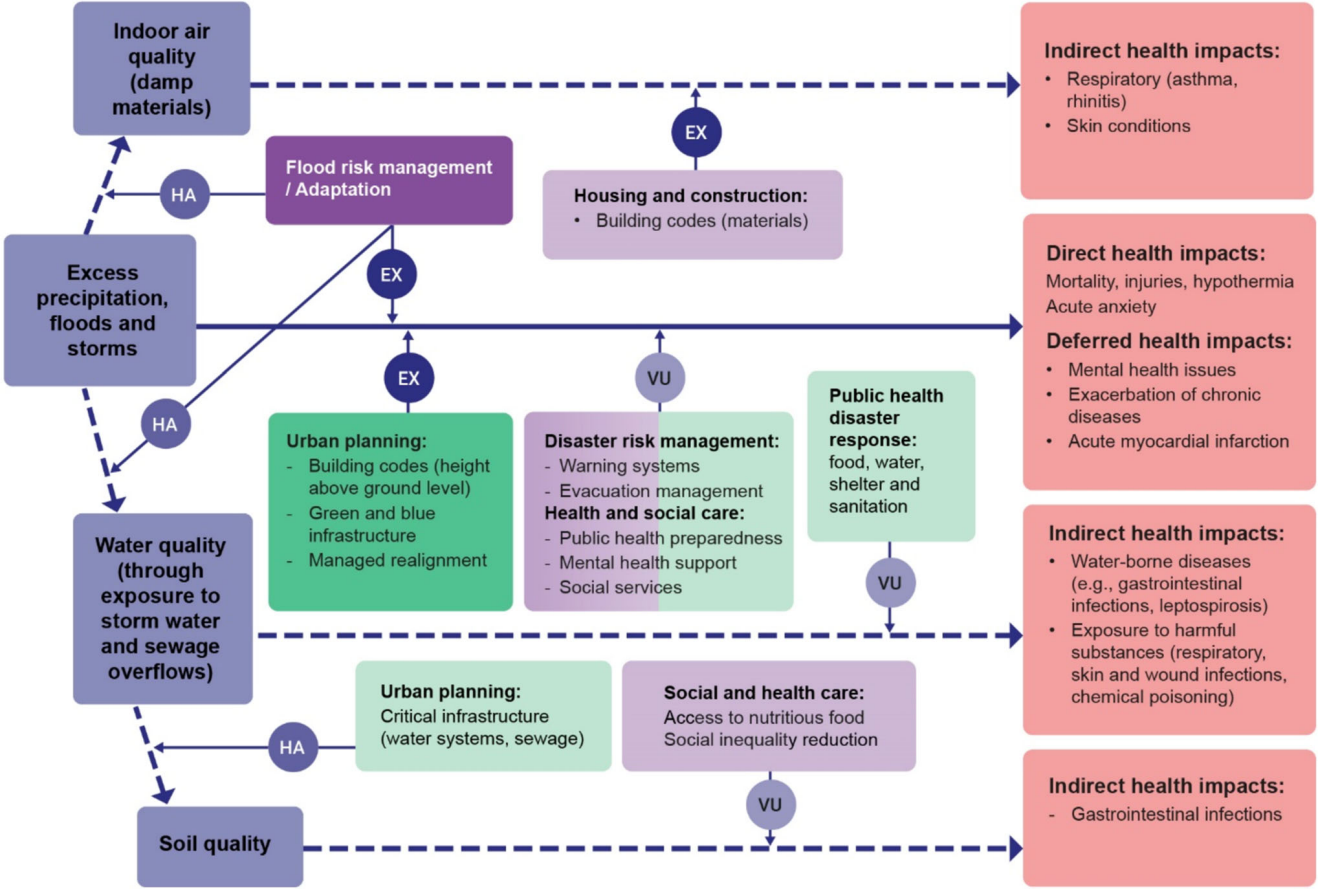
Floods and storms cause direct (increased mortality, injuries, distress) and deferred impacts (mainly related to mental health). Additionally, floods & storms cause disturbances in ecological systems through overflows and release of hazardous substances or pathogens into water and subsequently soil, causing indirect impacts on human health. Also, unhealthy levels of airborne moulds post-flood cause respiratory diseases in residents. All three categories of impacts are influenced by policies of high and low intentionality. Dark purple denotes *symbolic* policies, dark green denotes *concrete* policies, fair purple denotes *contiguous* policies, fair green denotes *contributive* policies. *VU* vulnerability, *EX * exposure, *HA * hazard. Dashed arrow denotes indirect impacts, solid arrow denotes direct and deferred impacts

Floods, storms, and increased precipitation cause three types of impacts on human health (see more details in Jurgilevich et al. (2023) and summary in Fig. 3). *Direct* impacts of floods and storms include mortality, injuries, acute anxiety, to name a few (Lane et al. 2013). Postponed or *deferred* impacts of these events include various mental health conditions (PTSD, chronic anxiety). Furthermore, a threefold increase in acute myocardial infarction has been registered and associated with post-Katrina, as people suffered from the effects of the storm, exacerbated by the loss of housing, employment, insurance, and increased substance abuse and medical non-compliance (Gautam et al. 2009). In addition to that, floods and storms cause overflows, which contaminate water and soil causing such *indirect* impacts to human health as gastrointestinal and other water-borne pathogen infections and exposure to hazardous substances (Lane et al. 2013; Padgham et al. 2015). Finally, damp materials and unhealthy levels of airborne moulds in the buildings affected by floods have caused a range of respiratory diseases among people living and working in those buildings (Hasegawa et al. 2015).

#### High intentionality policies (responses)

S*ymbolic* policies that reduce direct risks of floods and storms (drowning, hypothermia, acute anxiety, or injuries and morbidities following urban environment destruction or power outages) are related to adaptation, as well as flood protection/management strategies and plans (Fig. 3). These plans and strategies create space for *concrete* responses, most of which are targeted at reducing *exposure* through building codes or by modifying local topography and increasing the share of permeable surfaces with managed realignment, and green and blue infrastructure (Lane et al. 2013; Plag and Jules-Plag 2013; Sörensen et al. 2016; Berndtsson et al. 2019).

#### Low intentionality policies

Policies with low intentionality include mainly those moderating *vulnerability* in cases of direct risks from floods and extreme precipitation events (Fig. 3). *Contributive* policies enhance citizens’ *adaptive capacity* and include disaster preparedness and relief (Lane et al. 2013). Furthermore, vulnerability to the direct risks of flood can be decreased with public and mental health support and social services preparedness (Lane et al. 2013). Similar policies help to reduce *vulnerability* to the deferred risks of floods, such as post-traumatic stress disorder, anxiety, substance abuse and other conditions (Gautam et al. 2009; Lane et al. 2013). Additionally, there has been progress in examining how climate change and urban form exacerbate mental health conditions, post-traumatic stress disorder and emotional stress in vulnerable populations (Haney et al. 2010; Lane et al. 2013). Urban planning policies enhancing blue-green space, proportion of vacant lands and access to greenness moderate *sensitivity* to the deferred risks of floods (Hiscock et al. 2017; Pope et al. 2018) and are categorized as *contiguous.*

Storms and floods also cause indirect impacts on human health, which manifest through worsened indoor air (organic and inorganic air-transmitted particles from the damp or wet building materials) (Nazaroff 2013; Vardoulakis et al. 2015; Salthammer et al. 2018), and water and soil quality (resulting from overflows and release of hazardous substances). In addition to high intentionality flood adaptation policies that reduce hazard severity, also housing and construction policies are *contiguous* in reducing the *exposure *(Fig. 3).

*Contributive* policies reduce *vulnerability* to the indirect impacts of floods, such as water-borne diseases and exposure to harmful substances that can follow stormwater or sewage overflows (Charron et al. 2004; Lane et al. 2013; Paterson et al. 2018) (Fig. 3). These include disaster preparedness of public health in terms of the provision of food, shelter, water, and sanitation (Paterson et al. 2018). Furthermore, contamination and pathogens can get transferred to food through urban farming, which has been steered as an adaptation measure to cope with food shortage (Lwasa et al. 2015; Padgham et al. 2015). In this case, urban planning and critical infrastructure maintenance are *contributive* policies reducing the severity and probability of the *hazards* (overflows). Policies concerning the root causes of *vulnerability*, such as those creating or reducing social inequality or ensuring access to nutritious food, influence the severity and probability of risk and are categorized as *contiguous* (Dixon et al. 2007; Xu et al. 2019; Hashem 2020) (Fig. 3).

## Discussion and conclusions

Our analysis shows that within the current conceptualization of risk with response as intentional climate interventions (Simpson et al. 2021), many policies that influence risk emergence are being overlooked. It is apparent that many high intentionality policies, i.e., adaptation, are targeted at exposure and influence the hazard severity in the cases of indirect risks. At the same time, policies influencing vulnerability, are mainly those with low intentionality. These policies also address the root causes of vulnerability, including social justice, equity, cohesion, security, health, welfare, and neighbourhoods that affect physical and mental well-being (Anguelovski et al. 2018; Juhola et al. 2022). While it may not be the task of adaptation to restore past injustices or to address structural inequalities, it is important to recognize and prioritize these low intentionality policies that contribute to the prioritization of adaptation in places and for groups who need it the most. In addition, these low intentionality policies aimed at enhancing justice in adaptation can guide or be integrated with high intentionality policies, such as adaptation strategies and concrete adaptation policies, as is currently done in some cities. The city of Toronto, for example, places social justice as the central point of their climate resilience strategy by recognizing that social injustices are the root causes of climate vulnerability. Thus, the adaptation action in the strategy is planned in two ways: (1) pursuing transformative change by implementing policies targeted at reducing social injustices and climate vulnerabilities through welfare and equitable housing policies, and (2) by compensating and prioritizing vulnerable groups in their adaptation action (Toronto Resilience Strategy 2019).

The policies shaping indirect and deferred risks are mainly those with low intentionality. However, we also observe that indirect and deferred risks can be avoided, or their severity can be reduced through policies with high intentionality aimed at direct risks, in the first place. For example, flood management moderates the probability and severity of indirect risks, such as water contamination due to overflows, similarly to disaster response reducing vulnerability to the indirect risks associated with food and water poisoning, as well as to the deferred mental health risks (Gautam et al. 2009). Building on these observations, we note that the delineation and connection of different policies to the types of risk allows for a pathway approach to sequence and prioritize responses (Haasnoot et al. 2013). For example, exposure to the direct health risks to heat can be targeted with policies with high substantiality (i.e., concrete and contributive policies), which also reduce deferred risks of heat for outdoor workers. In cases of high exposure in the absence or failure of these policies, occupational healthcare (here a contiguous policy) can be sequenced to reduce vulnerability and the severity of the impact (Jurgilevich et al. 2023). Similarly, in cases of flood risks, policies with high substantiality (concrete and contributive), such as disaster risk management and flood risk management reduce direct health risks. Same time, public health preparedness (contributive) alleviates vulnerability and severity of deferred and indirect risks and adequate disaster relief providing safe food, water and shelter minimize indirect impacts through contaminated water and food (Lane et al. 2013). For example, studies have shown how the absence or failure of long-term disaster relief and social support policies have led to adverse mental and physical health outcomes post-Katrina (Gautam et al. 2009). Overall, these observations support the call for an integrated approach in urban sustainability transformations in addressing health risks by tapping into systemic effort across housing, infrastructure and construction and urban planning, in addition to social and healthcare (Crane et al. 2021).

This raises the question of the temporal dimension of both risk and of policy impact. With risk and vulnerability being dynamic (Ara Begum et al. 2022), it is critical to plan adaptation considering also the temporal dimension of policy outcomes. Many of the policies, especially low intentionality policies aimed at reducing vulnerability, for example, welfare, equity-promoting or social policies, take longer to achieve the expected outcome compared to technological or infrastructural responses. Therefore, low intentionality policies are important to include into risk assessments and adaptation with long-term horizons, which requires strategic approach to adaptation and overall urban planning and governance. Likewise, a disregard of these policies may result in unjust and inequitable adaptation both in the short-term and for the generations to come (see, e.g. temporal and intergenerational justice) (Goodin 2010; Teodoro et al. 2023).

The implications of our study stress the need for a more detailed discussion of what constitutes response and what policies contribute to vulnerability and risk emergence and development. Given that vulnerability does not develop in a social vacuum (Amorim-Maia et al. 2022), it is imperative to critically examine the policy context that shapes vulnerability through its root causes and how it is moderated through physical or social environment. The inclusion of low intentionality policies in addition to climate responses provides an entry point for adaptation research and practice that has a strong link to vulnerability research. Further examination of risk according to three different types (direct, indirect, and deferred) and connection of these with relevant policies and responses prompts a systems approach to adaptation considering risk complexity and temporal dynamics.

## Supplementary Information

Below is the link to the electronic supplementary material.

Supplementary file1 (PDF 168 KB)
