# Supplementary material for: Making sense of response: How policies affect climate vulnerability
Source: Ambio. 2025 Feb 3;54(7):1142–52. doi: 10.1007/s13280-025-02140-w (PMC12134250; doi:10.1007/s13280-025-02140-w)
Supplement: Supplementary file 1 — Supplementary file1 (PDF 168 KB) [file 13280_2025_2140_MOESM1_ESM.pdf]

Ambio

Supplementary information

This supplementary information has not been peer reviewed.

Title: **Making sense of response: how policies affect climate vulnerability**

Search and review strategy:

The examples have been collected with a non-systematic literature review, published in (Jurgilevich et al. 2023) using an iterative research strategy and snowball sampling. Based on the iterative research strategy, we searched literature with general queries (‘climate’ AND ‘urban’ AND ‘health’) and directed queries - specific hazards, urban and specific impacts on human health (e.g., ‘heat’ AND ‘urban’ AND ‘maternal health’) using Scopus, Google Scholar and Web of Science. Then, relying on snowball sampling principles (Johnson, 2014), we conducted new searches as new health impacts emerged from the literature. Next, we analysed the sample with a directed approach (Hsieh & Shannon, 2005) to identify policies and responses influencing risk and its determinants (hazard, vulnerability and exposure). As the primary and secondary academic literature is used here to exemplify responses and their interactions, we do not consider the proposed policy list to be exhaustive. Rather we point the directions for other studies in identifying responses for climate risk assessments. The analysis and categorisation has been done by three co-authors independently and then triangulated until the consensus has been reached.

References

Hsieh, H.-F., & Shannon, S. E. (2005). Three Approaches to Qualitative Content Analysis. *Qualitative Health Research*, *15*(9), 1277–1288. https://doi.org/10.1177/1049732305276687

Johnson, T. P. (2014). Snowball Sampling: Introduction. *Wiley StatsRef: Statistics Reference Online*. https://doi.org/10.1002/9781118445112.STAT05720

Jurgilevich, A., Käyhkö, J., Räsänen, A., Pörsti, S., Lagström, H., Käyhkö, J., & Juhola, S. (2023). Factors influencing vulnerability to climate change-related health impacts in cities - a conceptual framework. Environment International, 107837. https://doi.org/10.1016/J.ENVINT.2023.107837
